# Simultaneous dyeing and antibacterial finishing of polypropylene using vinyl sulfone dye under supercritical carbon dioxide

**DOI:** 10.1038/s41598-022-12680-w

**Published:** 2022-05-24

**Authors:** Tarek Abou Elmaaty, Abdalla Mousa, Hatem Gaffar, Heba Sorour

**Affiliations:** 1 Department of Textile Printing, Dyeing and Finishing, Faculty of Applied Arts, Damietta University, Damietta, 34512 Egypt; 2 Textile Research and Technology Institute, National Research Centre, 33 El Bohouth St, Dokki, Giza, 12622 Egypt; 3 Department of Material Art, Galala University, Galala, 43713 Egypt

**Keywords:** Soft materials, Chemistry, Chemical engineering

## Abstract

Polypropylene fibres are difficult to dye using commonly used techniques due to the high crystallinity and non-polar aliphatic structure, that lack reactive places for dyes in the molecule. Dyeing PP fabric in scCO_2_ with antibacterial dyes merged the dyeing and finishing methods, resulting in a more productive technique in terms of water and energy consumption. Unmodified polypropylene fabric was dyed with 4-[2-[4-(ethenylsulphonyl)phenyl]diazenyl]-*N*,*N*-diethylbenzenamine antibacterial dye under scCO_2_ medium. The influences of scCO_2_ working parameters, such as dye concentration, pressure, dyeing time, and temperature, on fabric dye absorption expressed as color strength were studied. The color strength (K/S) was measured as well as CIELAB color parameters. The results were compared with its water dyeing analogue and it was observed that color strength as well as color depth (L) of the samples dyed in scCO_2_ were noticeably better than its water counterpart. In both scCO_2_ and water, the fastness properties (washing, rubbing, and light) of the dyed samples were excellent. Antibacterial activity of the dyed polypropylene sample in scCO_2_ was estimated and the results indicated good antibacterial efficiency.

## Introduction

Polypropylene (PP) has various excellent properties, such as low density, good anti-static characteristics, high toughness and resilience, zero water adsorption, high tensile strength and excellent environmental resistance. PP is useful for home-furnishing and industrial applications^1,2^. Because of the low dyeability of conventional dyeing methods, its demand in clothing is limited^3^. Due to the high crystallinity and non-polar aliphatic structure of PP, which lacks reactive places for dyes in the molecule, the fibres are difficult to dye using traditional techniques.

Much research has been reported to overcome this problem^4–7^ and to improve the dyeability of PP fibers. The application of novel polymer additives^8^ changed the positive characteristics of PP fibers. Another method is using low temperature plasma technique to improve dyeability of PP^6^. Sahinbaskan *et. al.* studied the use of microwave energy for dyeing polypropylene^9^ while Toshniwal *et. al.* has produced nanocomposite PP by utilizing nanoclay modified with quaternary ammonium salt, which can be dyed with both acid and disperse dyes^10^. Although modifications of PP fabrics have made it probable to dye.

Water shortage has recently prompted the development of new technologies for textile chemical wet processing that use as little water as possible^11–14^. The use of scCO_2_ fluid (SCF) allows for an anhydrous dyeing process with numerous advantages. This substance, when highly compressed, can have the properties of both a liquid and a gas. Using supercritical carbon dioxide instead of water as a dyeing medium provides numerous environmental benefits in the textile industry, including zero waste emission, a high uptake rate, and low energy consumption^15,16^. Multiple research papers mentioned that scCO_2_ could be successfully used for the dyeing of natural and synthetic fabrics^17–23^.

Dyeing PP fabric in scCO_2_ with antibacterial dyes merged the dyeing and finishing methods. In view of our efforts to dye synthetic fabrics in scCO_2_^24–30^ especially PP, we have previously succeeded in developing a new method for dyeing PP fabrics with disperse dyes under aqueous and scCO_2_ medium. It was noticed that the color strength of the dyed PP fibres in scCO_2_ was found to be higher than in water. The fastness properties (washing, light, rubbing) of the dyed samples under study in scCO_2_ and water were excellent^31^. Sulfone derivatives provide an example of an important class of bioactive compounds. Literature described sulfone as anti-fungal^32^, anti-inflammatory^33^, anti-HIV^34^, anti-tubercular^35^, anti-cancer^36^, anti-hepatitis^37^ and anti-tumor^38^ agents.

In continuation of our work, we report here on a one- step dyeing and antibacterial finishing of PP fabrics with synthesized disperse reactive dye containing sulfonyl functional group under scCO_2_ medium.

## Experimental sections

### Materials and dyes

#### Dyes

**Figure 1:**
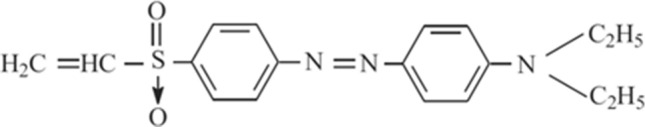
The chemical structure of the synthesized antibacterial vinyl sulfone disperse reactive dye.

The orange antibacterial vinyl sulfone disperse reactive dye (Fig. 1) was produced using the method previously mentioned and was prepared for use in scCO_2_^39^.

#### Material

The dyeing substrate was a 100% unmodified polypropylene fabric provided by Shikisen-sha Company (Osaka, Japan). Crystallinity of polypropylene fabrics is calculated using Eq. (1)

1 $${\text{ Crystallinity }}\left( \% \right) \, = \, \Delta {\text{H}}/\Delta {\text{Hm }}*{1}00.$$

Δ H = melting enthalpy: 105.8 J/ g,

Δ Hm = ideal crystal melting enthalpy: 209.1 J/g (Brandrup, 1999).

Crystallinity: 50.6%

### Water dyeing apparatus

Infra color dyeing system was used for water dyeing. It was composed of beakers mounted on a circular beaker carrier wheel. Heating with infrared radiation, cooling with air, and automation with the DC4 F/R microprocessor programmer. The maximum temperature reached 140 °C, with a maximum average heating rate of 5 °C/min and a maximum cooling rate of 3 °C/min.

### Supercritical carbon dioxide dyeing apparatus

**Figure 2:**
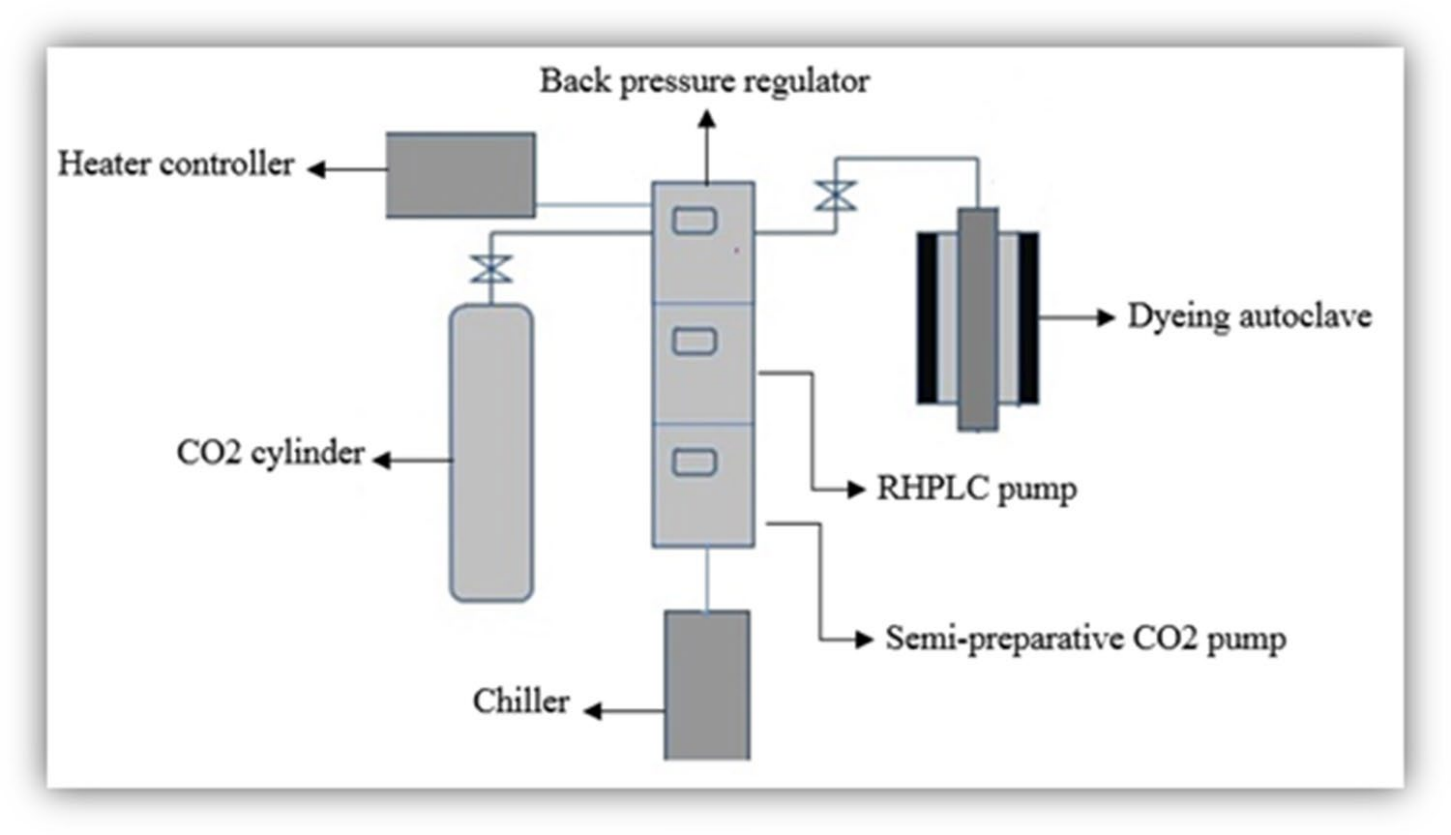
Supercritical carbon dioxide dyeing apparatus.

As shown in (Fig. 2), the main components of a scCO_2_ apparatus are: CO_2_ cylinder, a chiller (model Julabo FL601), an extraction RHPLC pump (model JASCO PU-4180), a semi-preparative CO_2_ pump (model JASCO PU-4386), a heater controller (model HC-2068–01), and a back pressure regulator (model JASCO BP-4340) with a maximum rate of pressure of 30 MPa, a temperature controller and speed controller (model EYELARCX-1000 H) with a maximum temperature rate of 130 °C, a dyeing autoclave with an internal capacity of approximately 50 mL, and a maximum CO_2_ flow rate of a circulation pump of 10 mL/min,

### Procedures

#### Dyeing unmodified PP fabrics in water

A Laboratory-Scale Thermal HT Dyeing system with a liquor-to-goods ratio of 1:50 was used for the dyeing experiment. The dyeing was performed at a concentration of 2% dye (based on the fabric weight). Then, in the dyeing machine, a PP fabric (10 × 10 cm) was inserted. Over the duration of 45 min, the temperature was increased from 35 °C to 130 °C, and dyeing was continued at that temperature for around 1 h. The samples were dyed, then washed in a bath containing 2% nonionic detergent (Sera Fast C-RD) at 60 °C, rinsed, and dried at room temperature.

#### Dyeing unmodified PP fabrics in scCO_2_

In the beginning, (CO_2_) gas was cooled to -5 °C by the chiller. PP fabric (3 × 10 cm) was covered by the screw tube and was inserted in the dyeing vessel. The dye was added to the bottom of the vessel's floor, and the concentration of dyestuff used ranged from 1 to 3%. (owf). Liquid CO_2_ was transported from the valve to the entire system by the semi-preparative CO_2_ pump. The autoclave was sealed and heated until the system reached the desired pressure (15, 20, 250 MPa), temperature (100, 120, 130 °C), and dyeing time (1, 2, 3 h) in line with the study of dyeing in scCO_2_. The shut-off valve was slowly opened to release CO_2_ until the pressure in the dyeing vessel reduced and reached atmospheric pressure automatically. The dyed sample was removed from the vessel. To eliminate the remaining dyestuff on the surface of the dyed samples, they were immersed in a bath containing 2% nonionic detergent (Sera Quick CRD) at 60 °C, rinsed with water, and dried at room temperature.

### Measurements and testing analysis

#### Color assessment

A spectrophotometer, Japan; model CM-3600 A, manufacturer KONICA MINOLTA, was used to measure the (K/S) and CIELAB color parameters (lightness (L*), Chroma (c*), hue (h*), the degree of redness (+ ve);greenness (-ve) (a*); the degree of yellowness (+ ve) and blueness (−ve).

(b*) of the dyed samples. The K/S values of dyed polypropylene fabrics were calculated using the Kubelka–Munk Eq. (2) at the wavelength of maximum absorption for vinyl sulfone dye.

2 $$K/S = \, \left( {1 \, - \, R} \right)^{2} /\left( {2R} \right)$$

The total color difference (∆E) was represented in terms of CIE LAB color space data. It was computed using Eq. (3)

3 $$E = ((L*_{2} + L*_{1} )^{2} + (a*_{2} + a*_{1} )^{2} + (b*_{2} + b*_{1} )^{2} )$$

#### Assessment of color fastness

An ISO standard method was used to analyze the dyed samples. The light fastness of the dyed PP samples was evaluated using an ISO 105-B02 Xenon arc lamp test with a standard blue scale (ISO 105-B02:2013 Part B02: Color fastness to artificial light: Xenon arc fading lamp test). The dyed PP samples were also analyzed for fastness to washing and rubbing according to ISO (International Organization for Standardization) 105-A03:1993 (ISO 105-A03:1993 Part A03: Grey scale for assessing staining) and ISO (International Organization for Standardization)105-A02:1993 (ISO 105-A02:1993 Part A02: Grey scale for assessing staining) and ISO (International Organization for Standardization)105-A02 (ISO 105-A02:1993 Part A02: Grey scale for assessing change in color).

#### Mechanical testing of unmodified PP fabrics

The mechanical properties of undyed and dyed PP fabrics were tested using a Tinius Olsen with a load cell 500 N, preload 0.01 N, speed 100 mm/min, and gauge length 100 mm in both scCO_2_ and water medium.

#### Antibacterial activity

Antibacterial activity assessment against *Staphylococcus aureus, Bacillus cereus, Escherichia coli, Pseudomonas aeruginosa* and expressed as zone of growth inhibition ZI (mm). The antibacterial activity was determined by the agar well diffusion method^12^.

#### Raman spectra of PP fabrics

Using the Jasco NRS-4500, the Raman spectra of undyed and dyed PP fabrics under scCO_2_ were observed in the range of 200 to 4000 cm^-1^.

### Statistical analysis

All of the experiments were completed by averaging three (sample) readings. The standard error of the mean was determined using the Eq. (4) below, and it was discovered to be + ( −) 0.1

4 $${\text{SE }} = \frac{S}{\sqrt n }$$

where SE is standard error, S is sample standard deviation, and n is the number of observations of the sample.

## Results and discussion

### Factors affecting dyeing properties of unmodified PP fabrics dyed in scCO_2_ medium

The influences of scCO_2_ working conditions, such as dye concentration, system pressure, dyeing time, and temperature were studied as influencing factors^40,41^. Values of the K/S of dyed samples were carried out in the range of (360–490 nm).

### Effect of dye concentration on K/S of unmodified PP fabric dyed in scCO_2_ medium

**Figure 3:**
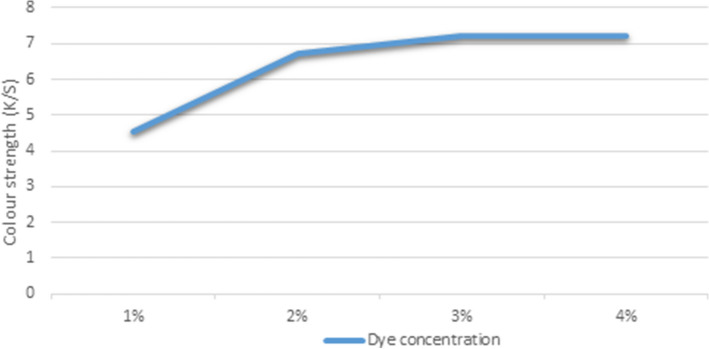
Effect of dye concentration on K/S of PP fabric.

At (1, 2, 3, 4%), the effect of dye concentration on (K/S) of PP fabric dyed in scCO_2_ was investigated. The dyeing pressure was set to 25 MPa, the temperature was set to 130 °C, the dyeing time was set to 60 min, and the dyeing medium flow rate was set to 7.500 ml/min during this procedure. As was revealed in (Fig. 3), the K/S of dyed samples in scCO_2_ was increased from 4.55 to 7.22 and it became constant at 4%. PP fibers have a high number of non-polar sites, which allows for multiple "Van der Waals forces" to occur, resulting in higher K/S values. The presence of alkyl chains in the structure of dyestuff made the materials more chemically alike and rely on the entropy of mixing, however, at 4%, the fabric was saturated and there is no longer increase in K/S.

### Effect of system temperature on K/S of unmodified PP fabric dyed in scCO_2_ medium

**Figure 4:**
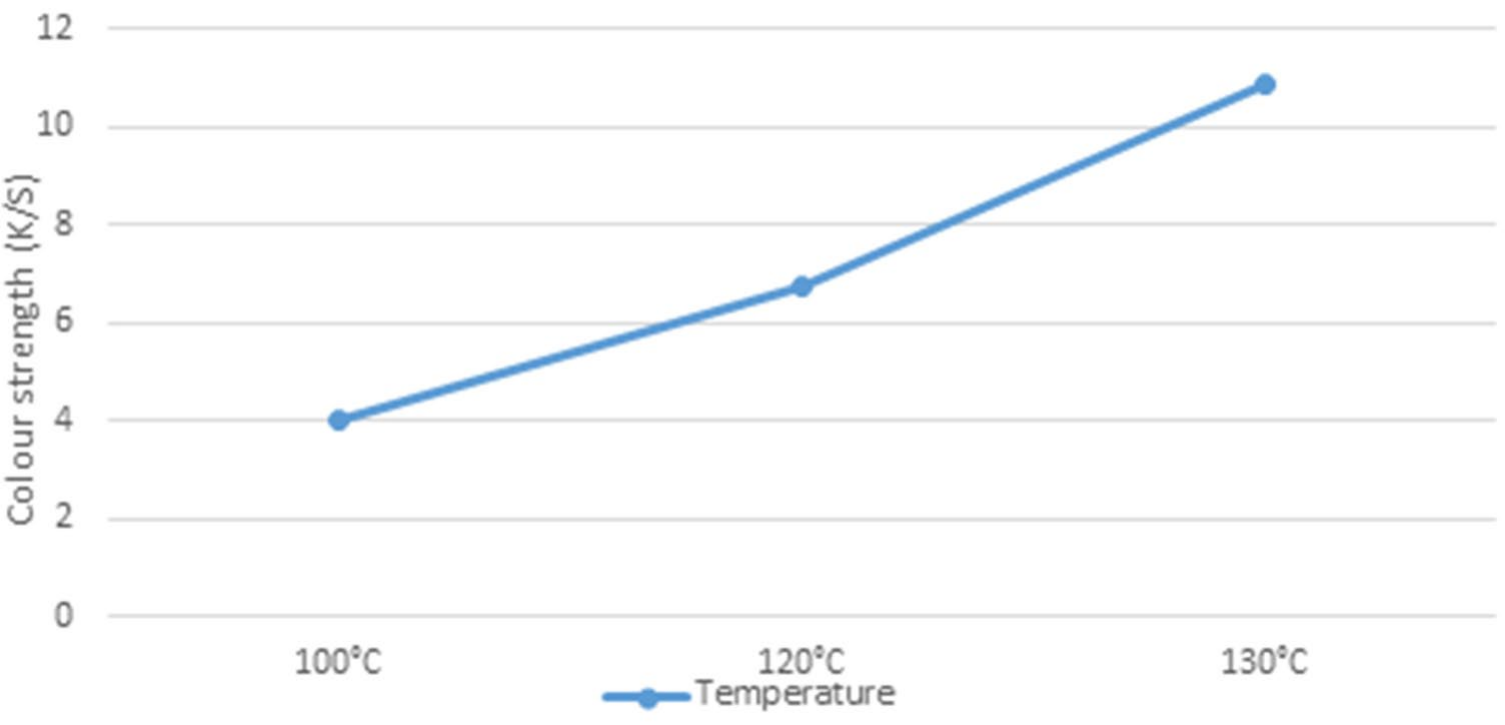
Effect of dyeing temperature on K/S of PP fabric.

The association between dye adsorption and system temperature (100,120, 130 °C) in scCO_2_ was studied as shown in (Fig. 4). The experiment was carried out at a 3% concentration, a pressure of 25 MPa, a dyeing time of 60 min, and a dyeing medium flow rate of 7.500 ml/ min. It was inferred that increasing the system temperature from 100 to 130 °C increased the K/S of PP fabrics dyed in scCO_2_. Increasing temperature increased the vapor pressure of the solute which lead to an increase in solubility. Additionally, the structure of the PP underwent swelling during dyeing due to high temperature and pressure which helps penetration of dye into the fiber. Also, the melting temperature of PP for most cases is about 160 to 170 °C depending on the tacticity and crystallinity of the polymer. Therefore, at 130 °C chain mobility was increased, and consequently aided the dyeing process. The result obtained was in good agreement with our previous work^31^.

### Effect of system pressure on K/S of unmodified PP fabrics dyed in scCO_2_ medium

**Figure 5:**
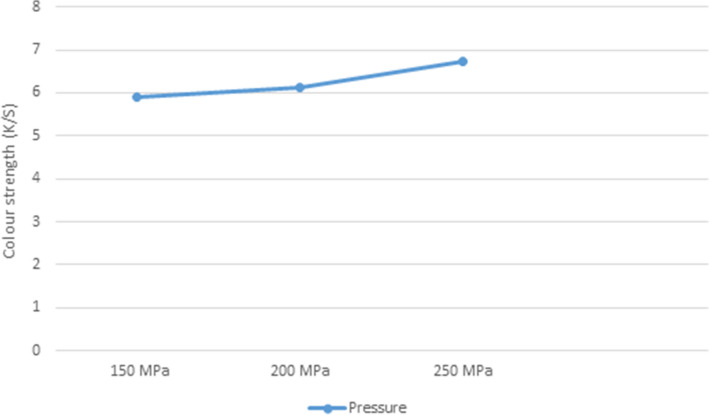
Effect of system pressure on K/S of PP fabric.

PP was dyed at a concentration of 3%, pressure varying from 15 to 25 MPa, a temperature of 130 °C, and a dyeing period of 60 min to investigate the effects of system pressure on the dye uptake of fibers. As shown in (Fig. 5), K/S values of dyed PP fabrics were improved by increasing system pressures to 15–25 MPa. Raising pressure increased the density of the scCO_2_ fluid, which improved the dye's solvent power and the interactions between the scCO_2_ medium and the intermolecular chains of the PP fibers in the dye bath^31^.

### Effect of dyeing time on K/S of unmodified PP fabric dyed in scCO_2_ medium

**Figure 6:**
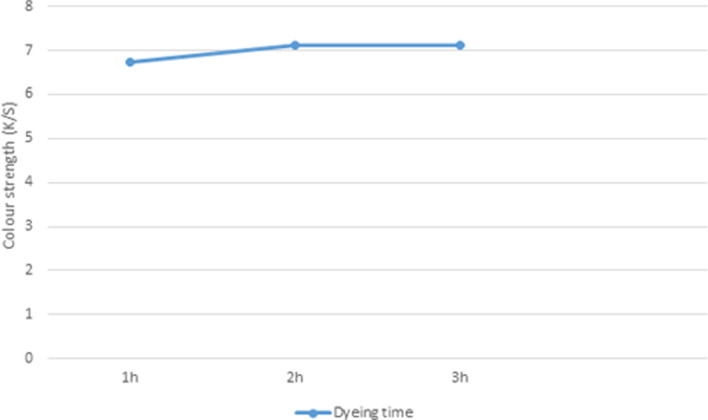
Effect of dyeing time on K/S of PP fabric.

At times ranging from 60 to 180 min, the effect of dyeing time on the adsorption of the vinyl sulfone disperse reactive dye on PP fabrics was observed. PP fabrics were dyed at 130 °C, a pressure 25 MPa, a dyeing medium flow rate of 7.500 ml/min. As presented in (Fig. 6), by increasing the time from 60 to 120 min, the K/S on PP fabric was increased, and it became constant at 180 min. This is due to low viscosity and high diffusivity of CO_2_ in supercritical state. As a result, it is much easier to permeate fiber than water during the dyeing process in scCO_2_. PP swells because of the permeation of CO_2_, thus causing a decrease in percent crystallinity and order which in turn improved dyeing. Therefore, the K/S values of the dyed PP were enhanced by increasing time.

### Color assessment

The reflectance data and color coordinates of supercritical dyed PP samples were measured using the CIELAB method in terms of L*, a*, and b* at a concentration of 3%, pressure 25 MPa, temperature 130 °C, and dyeing time 120 min. The color coordinates were listed in Table 1. which indicated that the dyes have good affinity to PP fabrics. Positive values of b* caused the color hues of dyes on PP fabrics to shift in a yellowish direction on the yellow-blue axis. The positive values of a* mean that the color hues of the dyes on PP fabrics changed in the redness direction on the red-green axis.

**Table 1:** CIELAB color parameters.

PP Dyed in scCO_2_	Average ∆E	k/s	L*	a*	b*	c*	h*
	68.18	6.74	64.47	31.16	52.33	60.9	59.23
PP Dyed in water	65.13	4	60.21	26.5	48.06	56.5	55.22

PP were dyed at a concentration of 3%, pressure 25 MPa, with a temperature 130 °C, a dyeing time 120 min.

Dyeing of unmodified PP fabric in scCO_2_, as a lower viscosity than water rendered of scCO_2_ capable of penetrating unmodified PP fabric thoroughly, hence resulted in a higher dye uptake compared with that using aqueous method with 3% dye concentration. In addition, the synthesized dye molecule containing an aliphatic substituent group (N, N-diethyl), which increase the solubility of the synthesized dye in scCO_2_, linking the dye with the unmodified PP fabric via the "Van der Waals forces"^43,44^. A comparison of color assessment of scCO_2_ and aqueous dyed fabrics showed that, without the addition of a carrier or dispersing agent, the appreciable color assessment of the dyed samples in scCO_2_ was significantly higher than that of the dyed samples in water.

The levelling properties of dyed unmodified polypropylene fabrics in scCO_2_ using the vinyl sulfone dye were illustrated in Table 1 from which it is clear that the average colour differences (ΔE, calculated from the CIE L*a*b* coordinates) of the dyed unmodified polypropylene fabrics indicated very good levelling properties in scCO_2_. This can be attributed to the fact that the structure of the unmodified polypropylene underwent swelling during scCO_2_ dyeing due to high temperature and pressure which is helping in penetration of vinyl sulfone dye into the fiber.

Color fastness, along with color strength, plays an important role in dyeing performance. According to the geometric grey scale, all of the dyed PP fabrics in water and scCO_2_ had excellent fastness values to washing, rubbing, and light, as shown in Table 2. The light fastness of samples dyed in scCO_2_ medium showed a good value (rating was 4) on the blue scale, which was evident. The results of rubbing fastness showed good values (rating between 3–4 and 4). The results of washing fastness in both water and scCO_2_ medium were outstanding. (rating between 4–5 and 5) as shown in Table 2.

**Table 2:** Color strength (K/S) and fastness properties of dyed PP fabrics using both scCO_2_ and water dyeing.

	Rubbing fastness		Wash fastness		Light fastness	K/S
	wet	Dry	Color change	Staining of adjacent fabric		
water dyeing	4	3–4	5	4–5	4	4
ScCO_2_	4	3–4	5	5	4	6.47

### Antibacterial activity

The antibacterial activity of the dyestuff under study and the dyed PP samples were investigated against *Staphylococcus aureus, Bacillus cereus, Escherichia coli, Pseudomonas aeruginosa* and expressed as zone of growth inhibition ZI (mm). The antibacterial activity was determined by the agar well diffusion method given in Table 3^24^. All tested dyestuff and treated PP samples showed positive antibacterial activities against the examined bacteria. The antibacterial activity of the unmodified PP fabrics dyed in scCO_2_ was significantly higher than that of the dyed fabrics in water. This can be attributed to the fact that the structure of the unmodified PP fabrics underwent swelling during scCO_2_ dyeing due to high temperature and pressure which helped penetration of more sulfonyl functional dye into the unmodified PP fabrics than that of the dyed fabrics in water. There was no discernible difference in the diameters of the inhibition zones for unmodified PP fabrics dyed in scCO_2_ before and after five washing cycles, confirming the durability of the PP fabrics dyed in scCO_2_.

**Table 3:** Diameter of zones of inhibition zone of the tested dye and polypropylene fabric against tested bacteria by the agar well diffusion method.

	*Escherichia coli*	*Staphylococcus aureus*	*Bacillus cereus*	*Pseudomonas aeruginosa*
ZI of dyestuff	14	18	28	15
ZI of dyed polypropylene fabric in water	7	10	18	9
ZI of dyed polypropylene fabric in scCO_2_	10	12	22	11
ZI of dyed polypropylene fabric in scCO_2_ (after 5 washing cycles)	10	12	22	11

PP was dyed at a concentration of 3%, pressure 25 MPa, with a temperature 130 °C, a dyeing time 120 min.

### Mechanical testing of unmodified PP fabrics

The results of elongation at break and maximum force (N) of the dyed fibers under both water and scCO_2_ medium were measured, and the results were reviewed in Table 4. After scCO_2_ dyeing there was an increase in elongation by 1.5% compared to undyed PP, while the sample dyed under water showed a decrease in elongation with a value of 8.5%. This was attributed to the fact that increasing temperature in water in presence of dispersing agent caused degradation of the polypropylene fabrics^26^ Alternatively, the sample dyed under scCO_2_ revealed a little decrease in maximum force by 7.4% while the sample dyed under water suffered from a 12% decrease. Ultimately, dyeing PP under scCO_2_ medium was better than dyeing in water medium because scCO_2_ dyeing maintained the physiochemical properties of the fiber.

**Table 4:** Mechanical test results of undyed and dyed PP fabrics.

Warp specimen	Elongation (%)	Maximum force (N)
Undyed sample	41.80	1214
Dyed sample in scCO_2_ at 120 °C	44.50	1160
Dyed sample in scCO_2_ at 130 °C	43.32	1130
Dyed sample in water	33.22	950

PP were dyed at a concentration of 3%, pressure 25 MPa, a dyeing time 120 min.

### Raman spectra

**Figure 7:**
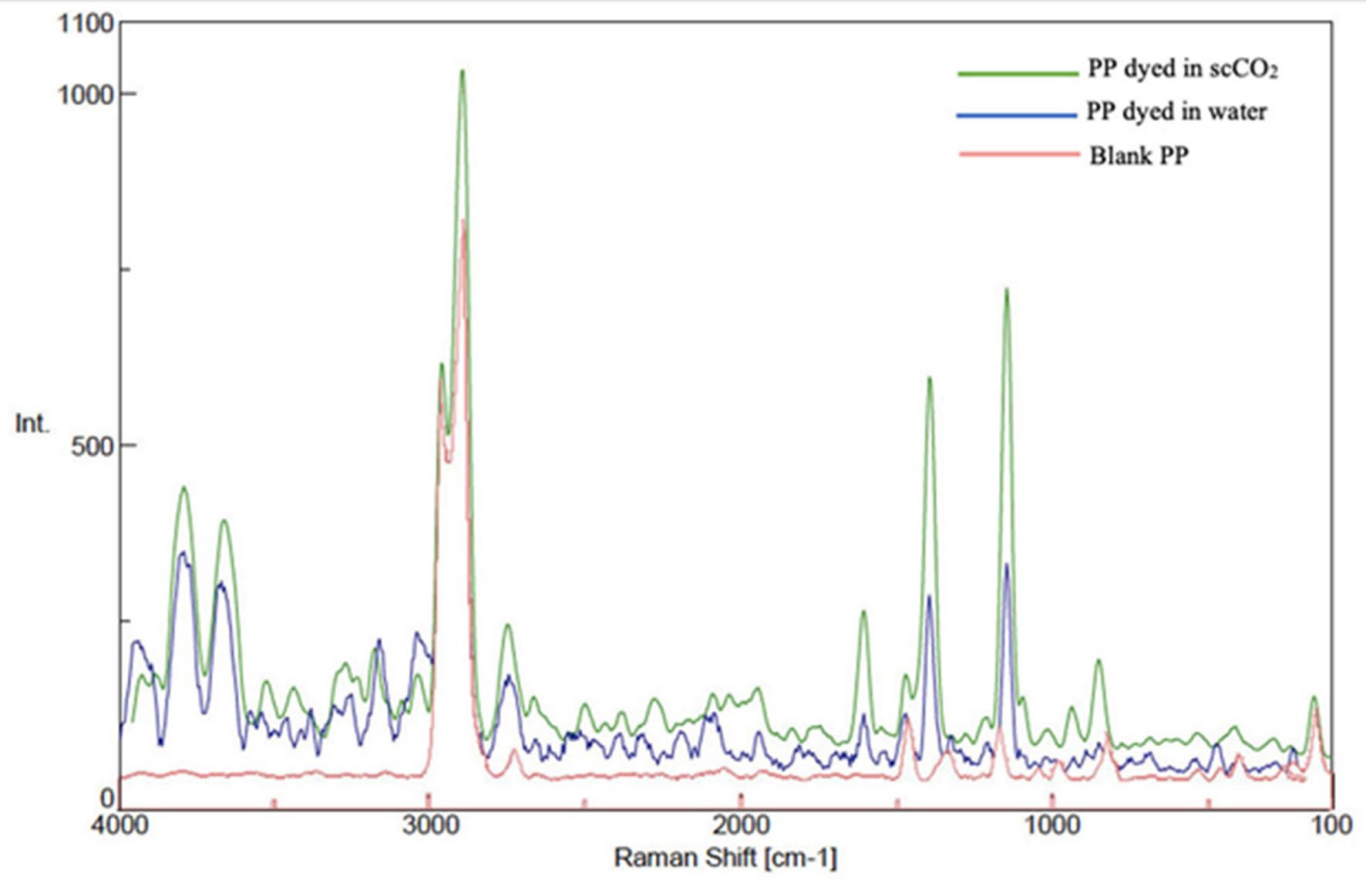
The Raman spectrum for undyed and dyed polypropylene in scCO2 and water.

**Figure 8:**
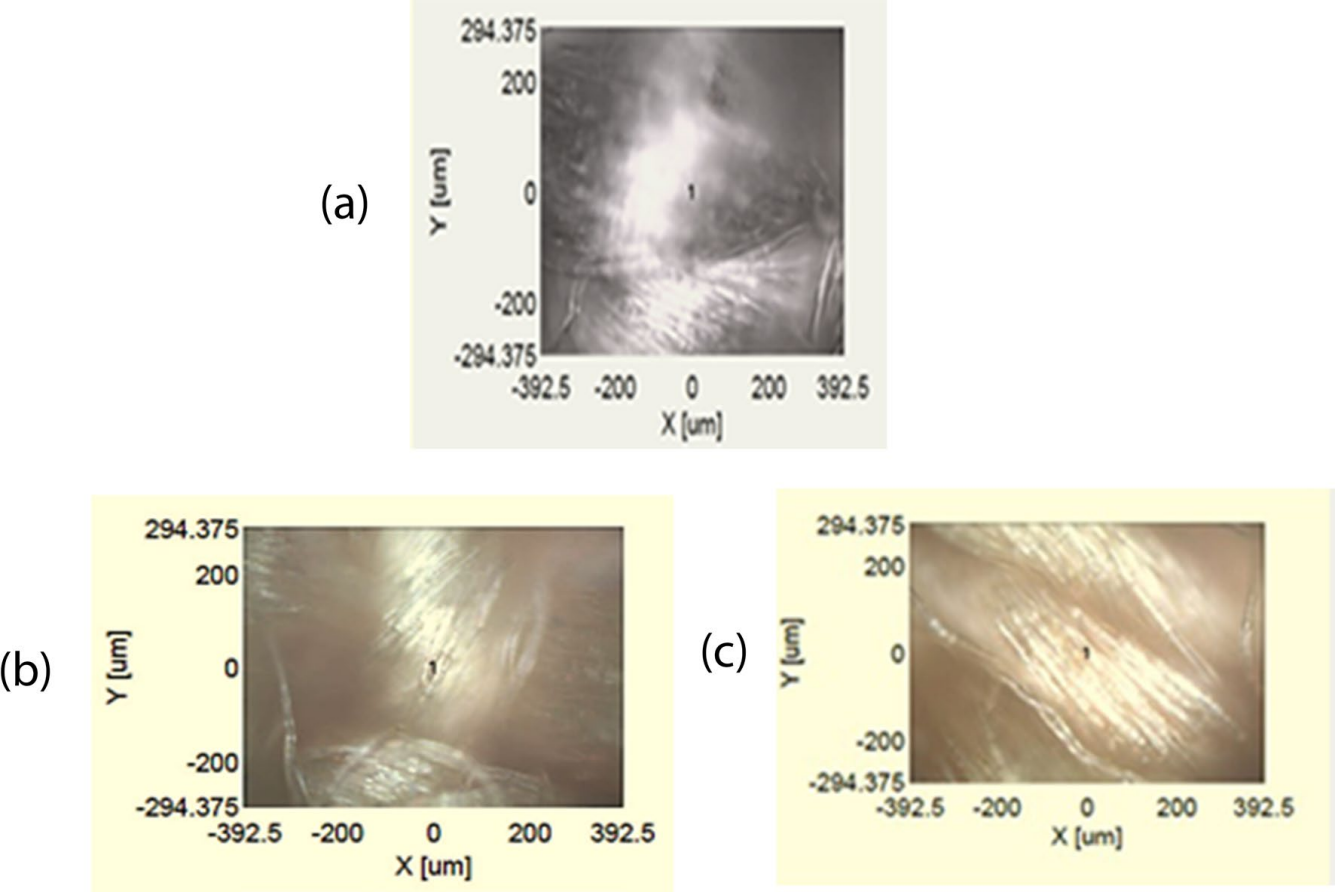
The Raman pictures for dyed and undyed polypropylene in scCO2 and water (**a**) blank PP (**b**) PP dyed in water (**c**) pp dyed in scCO2.

Figures 7 and 8 showed the Raman spectrum and pictures of undyed and dyed PP. A Raman spectrum for undyed PP indicated several peaks. First set of peaks were observed between 900 and 1600 cm^-1^. The rest of peaks were located between 2700 and 4000 cm^-1^. The similar trend of PP spectrum was studied by *Bhattacharyya *et al.^42^.

The vinyl sulfone disperse reactive dye under study was characterized by Raman analysis and to make sure that the dyeing happened to have absorption inside or on surface of the PP fabrics. A comparison of Raman spectra for dyed and undyed PP fabrics was given in Fig. 7. Spectra were collected in the range of (100 to 4000 cm^-1^). The spectra were acquired using scan time settings of (3 s) and a resolution of 2.32 cm^-1^/ pixel for fiber analysis. Figure 7 showed Raman spectra of the undyed and dyed samples of PP fabrics where the waves assigned as follows: 1470.2 cm^-1^ C-H, the absorption bands at 1325–1185.8 cm^-1^ (-N = N-), 3045.2 cm^-1^ (-C = C-), 1145.2 cm^-1^ (SO_2_). The recorded Raman spectra confirmed that all the designed and expected characteristic groups were implicated in the chemical structure of the vinyl sulfone disperse reactive dye.

## Conclusion

A facile one step dyeing and finishing process was constructed to dye unmodified PP fabrics with antibacterial disperse reactive dye in scCO_2_.The results showed that, the synthesized antibacterial vinylsulfone disperse reactive dye have high affinity for dyeing PP fabrics in water and scCO_2_. The K/S was enhanced using scCO_2_. The ratings of fastness to washing, light and rubbing ranged from very good to excellent with the increase of the depth of shade. The optimum dyeing conditions for dyeing PP in scCO_2_ were set to be 130 °C, pressure 25 MPa, dyeing concentration 3% at 120 min, based on the results of color strength, color fastness, and mechanical properties. Therefore, dyeing of PP fabric under scCO_2_ medium was proved to be effective and potential for industrial applications.

## Supplementary Information

Supplementary Information.

## Data Availability

All data generated or analyzed during this study are included in this published article [and its supplementary information files].
