# Supplementary material for: Simultaneous dyeing and antibacterial finishing of polypropylene using vinyl sulfone dye under supercritical carbon dioxide
Source: Sci Rep. 2022 May 24;12:8789. doi: 10.1038/s41598-022-12680-w (PMC9130219; doi:10.1038/s41598-022-12680-w)
Supplement: Supplementary file 1 — Supplementary Information. [file 41598_2022_12680_MOESM1_ESM.docx]

**Simultaneous dyeing and antibacterial finishing of polypropylene using vinyl sulfone dye under supercritical carbon dioxide**

**Tarek Abou Elmaaty*^a,c^, Abdalla Mousa^b^, Hatem Gaafar^b^, Heba Sorour^a^**

^a^ Department of Textile Printing, Dyeing& Finishing, Faculty of Applied Arts, Damietta University, 34512 Egypt.

^b^Textile Research and Technology Institute, National Research Centre, 33 El Bohouth St, PO 12622, Dokki, Giza, Egypt

^c^ Department of Material Art, Galala University, Galala 43713, Egypt.

*Corresponding author: tasaid@gu.edu.eg


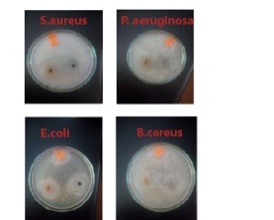


Fig 1. Diameter of zones of inhibition zone of the tested dye and polypropylene fabric against tested bacteria by the agar well diffusion method
